# Endoplasmic Reticulum-Associated Degradation-Dependent Processing in Cross-Presentation and Its Potential for Dendritic Cell Vaccinations: A Review

**DOI:** 10.3390/pharmaceutics12020153

**Published:** 2020-02-13

**Authors:** Jun Imai, Sayaka Ohashi, Takahiro Sakai

**Affiliations:** Laboratory of Physiological Chemistry, Faculty of Pharmacy, Takasaki University of Health and Welfare, Takasaki, Gunma 370-0033, Japan; 0621021@takasaki-u.ac.jp (S.O.); sakai@takasaki-u.ac.jp (T.S.)

**Keywords:** dendritic cell, cross-presentation, major histocompatibility class I, endoplasmic reticulum-associated degradation, molecular chaperone, DC vaccination

## Abstract

While the success of dendritic cell (DC) vaccination largely depends on cross-presentation (CP) efficiency, the precise molecular mechanism of CP is not yet characterized. Recent research revealed that endoplasmic reticulum (ER)-associated degradation (ERAD), which was first identified as part of the protein quality control system in the ER, plays a pivotal role in the processing of extracellular proteins in CP. The discovery of ERAD-dependent processing strongly suggests that the properties of extracellular antigens are one of the keys to effective DC vaccination, in addition to DC subsets and the maturation of these cells. In this review, we address recent advances in CP, focusing on the molecular mechanisms of the ERAD-dependent processing of extracellular proteins. As ERAD itself and the ERAD-dependent processing in CP share cellular machinery, enhancing the recognition of extracellular proteins, such as the ERAD substrate, by ex vivo methods may serve to improve the efficacy of DC vaccination.

## 1. Introduction

Cancer vaccination is the most well-studied immunotherapeutic strategy among cancer immunotherapies [1]. Like virus vaccines and toxoid vaccines, cancer vaccines immunize patients with proteins from cancer cells with the hope of activating the immune system to destroy the cancer cells. Cancer vaccines are intended to activate the response of cancer-specific cytotoxic T lymphocytes (CTLs), resulting in the rejection of cancer cells by long-lasting anti-cancer immunity. While anti-virus vaccines, such as the human papilloma virus (HPV) vaccine and the hepatitis B virus (HBV) vaccine, successfully prevent specific cancers caused by viruses [2], most cancer vaccines have failed or had a limited effect in clinical trials [1]. This limited effect is partially due to malignant cancer cells exhibiting weak immunogenicity, allowing for efficient immune escape [1]. Additionally, although cancer vaccines can activate cancer-specific CTLs, malignant cancer cells are equipped with several methods to evade the immune system [3].

To stimulate the cancer-specific immune response more effectively, dendritic cell (DC) vaccines were developed with high expectations, since DCs exhibit a strong ability to activate a cytotoxic response toward specific antigens [1]. DCs are isolated from the patient for immunotherapy, immunized with a cancer antigen or cancer lysate, and transfused back to the patient [1]. DCs internalize immunized proteins and present processed antigenic peptides to the major histocompatibility complex (MHC) class I (MHC I) and MHC class II (MHC II) molecules, which are presented via MHC II in other antigen-presenting cells (APCs) [1]. These specific activities of DCs are referred to as cross-presentation (CP), and play a definitive role in initiating CD8^+^ T cell-induced immune responses against cancer and/or viruses (cross-priming) or to induce peripheral tolerance (cross-tolerance) [4,5,6,7,8]. Since effective activation of cancer-specific CTLs results in the successful inhibition of malignant cancer progression [9,10], the effective CP of cancer-associated antigens is one of the essential requirements for an effective immune response in cancer immunotherapy [11,12,13]. However, in the absence of CP, the immune system theoretically produces predominantly T helper 2 (Th2) responses rather than T helper 1 (Th1) responses associated with antigen-specific CTLs, resulting in no tolerance to cancer. However, the results of DC vaccination have been disappointing, and limited CP activity may have resulted in insufficient numbers of CTLs [1].

In the last couple of decades, numerous efforts have been made to elucidate the molecular mechanism of CP, which revealed that immunized proteins are processed by the endoplasmic reticulum-associated degradation (ERAD) pathway [14]. ERAD was first described as a part of the cellular pathway for protein quality control in the ER: The unfolded protein response (UPR) [15]. Though the substrates of ERAD are unfolded proteins in the ER, these proteins are not degraded in the ER lumen, but rather retro-transported out of the ER lumen into the cytosol and degraded by the ubiquitin-proteasome system (UPS) [16]. While one of the aims of these investigations was the improvement of CP efficiency, which was partially accomplished in a mouse model [17,18,19], this has not contributed to the improvement of DC vaccination in clinical trials [20].

In contrast to investigations on the molecular mechanism of CP, deciphering the immune escape mechanism of malignant cancers has led to the establishment of new immunotherapeutic methods: Namely, immune checkpoint inhibition therapies [21,22,23,24]. However, CP by DCs is essential for the successful outcome of these methods [25]. In that sense, the DC vaccine appears to be an attractive cancer immunotherapy approach in combination with immune checkpoint inhibition therapy [26]. Additionally, recent research revealed that even in cancer chemotherapy or cancer radiation therapy, CP by DCs is essential in eliminating cancers [27,28]. However, insufficient CP efficiency persists through the rate-determining steps, not only in DC vaccination, but also for other cancer therapies. Therefore, CP efficiency has been described as the rate-determining step for these therapies, since poor CP efficiency results in the poor activation of cancer-specific CTLs. Several rate-limiting steps have been shown to critically contribute to CP efficiency:

(i) Restricted lysosomal degradation of extracellular proteins
(ii) Recruitment of ERAD-related molecules into endocytotic compartments
(iii) Retro-transport of extracellular proteins into the cytosol

In this article, we discuss the current concepts of CP, focusing on the improvements of CP efficiency, and more specifically, on the cellular transport route employed by immunized proteins and the molecular mechanism of their recognition as the ERAD substrate.

## 2. Dendritic Cell (DC) Vaccination

### 2.1. DC Subsets

DCs are a diverse group of specialized APCs with key roles in the initiation and regulation of antigen-specific immunity and tolerance [29]. In the peripheral tissue, DCs incorporate extracellular antigens and sense their environments by cytokine receptors or pattern recognition receptors (PRRs), which specifically detect pathogen-associated molecular patterns (PAMPs) or damage-associated molecular patterns (DAMPs) [30]. After detecting immunological stimulants and processing antigens, DCs undergo maturation and migrate into the draining lymph nodes where they demonstrate substantial morphological alternations, and initiate immune responses by presenting antigens to T cells and by providing immuno-modulatory molecules and cytokines according to the situation [31,32]. 

Both mouse and human DCs are divided into four main populations: conventional DCs (cDCs) [33], plasmacytoid DCs (pDCs) [34], monocyte-derived DCs (moDCs) [35,36], and Langerhans cells (LCs), all of which differ in ontology, phenotype, and functions. cDCs are further divided into two subpopulations called Th1-activating cDCs (cDC1s) and Th2-activating cDCs (cDC2s) in mice [31]. Closely related counterparts are found in humans; however, surface molecules and cytokine profiles do not precisely correspond to each other [37] (Table 1). cDC1s play indispensable roles in cancer immune therapy and have demonstrated the most substantial CP efficiency, irrespective of the protein uptake route [38]. In addition to their ability to activate CD8^+^ T cells via CP, cDC1s also initiate CD4^+^ helper T-cells for full activation of CTLs [39,40] and expansion of memory T cells [41]. Additionally, human (to a lesser degree) and mouse cDC1s produce IL-12, which is critical for the differentiation of naïve T cells into Th1 cells [42,43]. Indeed, expression of CP-related molecules was found to be higher in splenic cDC1s than in splenic cDC2s [44]. Cancer graft experiments in mice revealed that immune checkpoint inhibition therapies showed no effects without cDC1s [11,45]. Likewise, in humans, the migration of cDC1s in the cancer microenvironment is essential for immune checkpoint inhibition therapy [46]. These results indicate that cDC1s are the best-suited subset for DC vaccination; however, collecting an adequate number of human cDC1s remains challenging, resulting in too few for administration in a DC vaccination platform. While cDC2s are a potent inducer of Th2 and T helper 17 (Th17) responses, showing restricted CP ability in mice [35,38], human cDC2s can induce the polarization of diverse subsets of CD4^+^ T cells and activate CD8^+^ T cells to induce Th1 responses [47,48,49,50]. The CP efficiency of cDC2s was equivalent to that of cDC1s for receptor-mediated endocytosis, but was less efficient for pinocytosis and phagocytosis in mice [38]. Thus, cDC2s may be of use in DC vaccination; however, they are principally not recommended.

While pDCs produce large amounts of type I interferon and play central roles in the immune defense against viral infections [51], these cells show generally poor CP ability [52]. In both humans and mice, only myeloid-derived pDCs can activate CD8^+^ T cells, yet they exhibit relatively poor priming capacity for naïve T cells [47,48]. Therefore, pDCs cannot play a decisive role in DC vaccination.

The moDCs, which express CD11c and MHC II [53], differentiate from monocytes, predominantly under inflammatory conditions. Thus, they are also called inflammatory DCs [54,55,56]. Bone marrow-derived DCs (BMDCs) show similar characteristics to moDCs, which differentiate from myeloid cells under the presence of granular colony-stimulating factor (GM-CSF). Both moDCs [57,58] and BMDCs [59,60] show efficient CP capacity to activate naïve CD8^+^ T cells, similar to cDC1s. Further, in mice, they efficiently activate naïve CD8^+^ T cells for pinocytosis [61], less efficiently for receptor-mediated endocytosis, and not at all for phagocytosis [38]. Additionally, these cells can differentiate CD4^+^ T cells toward Th2 cells and Th17 cells in addition to Th1 cells in humans [49]. While moDCs and cDC2s are clearly distinguishable in mice, they express notably similar cell surface markers in humans. Currently, the precise ontology and specific roles played by these cells have not yet been elucidated in humans [31,37,50]. However, moDCs can be induced from blood monocytes in vitro using GM-CSF, and thus are not difficult to collect in high numbers in contrast to cDC1s [62]. Monocytes are also able to differentiate into myeloid-derived suppressor cells (MDSCs), or MDSC-like suppressor cells in some cases, which suppress anti-cancer immunity in both humans and mice [63]. These results indicate that further research is required to elucidate the prospects of human moDCs for DC vaccines.

LCs express langerin, a C-type lectin receptor localized in the epidermis, the outermost layer of the skin [64]. Langerin specifically binds with glycoconjugates, such as the high-mannose structure mannan or the β-glucan on the surface of pathogens [65]. LCs show a strong ability to capture, uptake, and process skin pathogens, as well as epidermal self-antigens. Moreover, LCs have the capacity to induce CD4^+^ T cell responses after migrating to secondary lymphoid tissue [66,67]. They also show a limited CP ability to activate CD8^+^ T cells in mice [68] and in humans langerin-dependently [69]. Since the skin is actively used as a vaccination site, LCs may play a role in specific DC vaccination platforms. Most of these DC classifications have been examined in mice, strongly indicating that experimental confirmation is required for DC vaccines [31,33].

### 2.2. DC Vaccination

As DCs are one of the rarest populations among leukocytes, DC precursors from patients are differentiated into DCs for DC vaccines. moDCs are the most commonly used DC subset in DC vaccination. Peripheral blood mononuclear cells (PBMCs) collected from a patient are differentiated into moDCs in the presence of GM-CSF and IL-4 [1]. BMDCs are also used, but not as frequently as moDCs. CD34^+^ precursor cells are harvested from the bone marrow of patients after administration of GM-CSF; cultured in the presence of GM-CSF, Fms-like tyrosine kinase 3 ligand (Flt3L), and TNF-α; and differentiated into a mixture of moDCs, cDCs, and LCs [1]. Likewise, the expansion of cDC1s from circulating BDCA-1^+^ DCs by Flt3L [72] or the isolation of primary circulating CD1c^+^ blood DCs [73] is possible, but the populations of circulating DCs represent less than 1% of PBMCs. Since cDC1s show the most potent ability to initiate CTL immunity against tumors in mice [74], cDC1s are highly recommended for DC vaccines [71]. While the availability of DCs shows marked variability with the aforementioned methods, the preparation method for DCs must be selected by considering both the reliability and efficiency of the harvested DCs. Recently, it was reported that cDC1-like cells were differentiated from human induced pluripotent stem cells (ipDCs) [75]. These cells might be applicable in DC vaccination in the near future. 

There are several antigen-loading methods for DC vaccines: antigenic peptides, tumor-associated antigens (TAA), DNA of TAA, viral vectors with TAA insertion, tumor lysates, and tumor-derived mRNA [1]. Though pulsing DCs with antigenic peptides is the most comprehensive method, information about the patient’s haplotype and tumor-specific antigenic peptides is indispensable [1]. This is also the case for TAA, DNA of TAA, and viral vectors with TAA insertion; the presence of antigenic peptides suitable for the patient’s haplotype must be confirmed before loading [1]. In early trials of DC vaccination by TAA, tumor-specific CTL responses were detected [76], indicating that successful CP induced tumor-specific CTLs. While the exact mechanism that accelerates CP efficiency is not yet established, Sipuleucel-T, the only Food and Drug Administration (FDA)-approved therapeutic cancer vaccine, provoked tumor-specific CTL responses against prostate cancer and resulted in better survival rates compared with the controls [77]. These results strongly suggest that the improvement of CP plays a significant role in the results of DC vaccination. Nevertheless, most TAA vaccines were able to induce a tumor-specific CTL response; in most cases, they showed some ability to induce tumor regression when administered alone. Thus, tumor cells appear to be equipped with capabilities to evade the immune system and it is difficult to only destroy these tumor cells by CTLs; thus, the successful combinatorial therapies, such as combinatorial therapies with the immune checkpoint inhibition therapies, are necessary. In this regard, total tumor lysates and tumor-derived mRNA are beneficial, as they do not require information about the patient’s haplotype and inevitably maintain unknown novel tumor antigens [1]. Additionally, the loading of total tumor lysates could neutralize immune-suppressive signals by regulatory T cells (Tregs), which is one of the immune avoiding systems of malignant tumor cells, through activating self-reactive, pro-inflammatory T cells (anti-Tregs) in humans [78]. From a practical standpoint, the purification of mRNA is not convenient compared to the preparation of tumor lysates. Furthermore, electroporation, which is the most efficient method to introduce mRNA into DCs, significantly reduces the number of DCs, which is already limited. These results indicate that the loading of total tumor lysates is an acceptable method when CP is efficient.

CP abilities vary among different subsets and uptake routes of extracellular antigens. Additionally, the particle size of the antigen-loaded beads also affects the CP efficiency of the same antigen in mice [79]. cDC1s showed the most extensive CP efficiencies for all protein uptake routes, such as receptor-mediated endocytosis, pinocytosis, and phagocytosis [38]. Murine cDC2s exhibit an effective CP ability similar to that of cDC1s for receptor-mediated endocytosis, but CP is less efficient for the other two uptake routes [38]. The CP efficiencies of murine moDCs were equivalent to those of cDC1s for pinocytosis, less efficient for receptor-mediated endocytosis, and not detectable for phagocytosis [38]. As mentioned above, LCs only show CP for receptor-mediated endocytosis. These differences indicate that the loading methods of tumor lysates must be optimized according to the DC subsets in DC vaccination.

## 3. ERAD-Dependent Processing in CP

### 3.1. Two Pathways for Cross-Presentation (CP)

**Figure 1:**
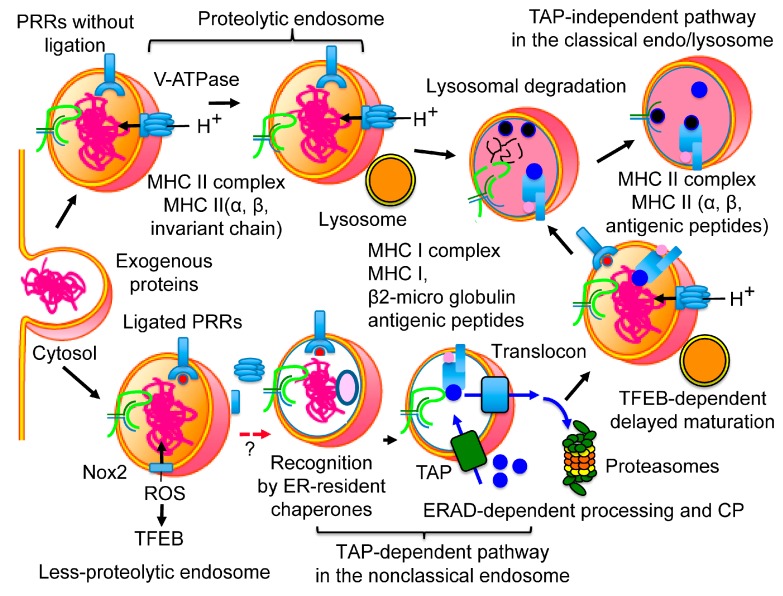
Maturation of endosomes and cross-presentation (CP). After internalization, exogenous proteins are transported into the endosome. Endocytic compartments without danger signals are acidified, fused with the lysosome, and designated as the proteolytic endosome. Since extracellular proteins without danger signals are self-proteins, those proteins are quickly degraded by the lysosomal proteases, which results in lower CP efficiency. When danger signals are detected by pattern recognition receptors (PRRs) (TLR2, TLR4, Nod1, Nod2), the acidification of endocytic compartments is delayed by the incomplete assembly of V-ATPase and the activation of Nox2, and resulted in the less-proteolytic endosome. The activated Nox2 produces reactive oxygen species (ROS), and ROS promote nonspecific disulfide bond formation to unfold extracellular proteins. As extracellular proteins with danger signals are non-self-proteins, these proteins are recognized by the endoplasmic reticulum (ER)-resident molecular chaperones in the non-classical endosome. Recognition by the ER-resident molecular chaperones accelerates ER-associated degradation (ERAD)-dependent processing of exogenous proteins and results in higher CP efficiency. ROS also activate TFEB, and activated TFEB promotes the maturation of the less-proteolytic endosome with a time difference. The activated TFEB also induces maturation of DCs by initiating the transcription of costimulatory molecules, several cytokines, and chemokines, including IL-1β, IL-6, TNF-α, and CCL5. In contrast to the major histocompatibility complex (MHC) I, MHC II presents antigenic peptides processed by lysosomal protease. MHC II associates with antigenic peptides under acidic conditions in the lysosome.

Two major pathways involved in the molecular mechanisms of CP are described: the transporter associated with antigen processing (TAP)-dependent pathway and the TAP-independent pathway [80,81,82,83,84]. In the TAP-dependent pathway, extracellular proteins are retro-transported into the cytosol through the cellular membrane and processed by the UPS [83,84] (Figure 1). The derived antigenic peptides are transported through the cellular membrane, trimmed, and loaded on MHC I with the aid of the peptide-loading complex (PLC) [85,86,87]. In contrast, in the TAP-independent pathway, extracellular proteins are processed by the lysosomal protease, cathepsin S, and loaded upon MHC I within the endo/lysosomal compartment by a peptide exchange reaction independent of TAP [88] (Figure 1). While the TAP-independent pathway has some functions in CP, recent investigations have shown that the TAP-dependent pathway is a major pathway required for CP to regulate cell-mediated immunity. Consequently, immunoproteasome-deficient BMDCs showed impaired CP ability in vitro, as well as in vivo, in a mouse model [82]. The cytoplasmic delivery of exogenous proteins enhanced CP efficiency in a murine model [89]. Expression of PLC was higher in splenic cDC1s than in splenic cDC2s [44], and cDC1s were responsible for both cross-priming [44] and for cross-tolerance [90]. In addition to these two pathways, several other CP-related pathways were found in DCs [91,92]. However, these pathways do not contribute significantly to the ability of CP to initiate naïve CD8^+^ T cells and thus will not be discussed further in this review.

### 3.2. Molecular Mechanism of the TAP-Dependent Pathway

#### 3.2.1. Protection of Extracellular Proteins from Lysosomal Degradation

In the TAP-dependent pathway, extracellular proteins are protected from lysosomal proteases and are processed by the UPS [93,94,95]. To accommodate this, DCs are equipped with several molecular machineries to protect extracellular proteins from rapid lysosomal degradation. First, DCs express lower amounts of lysosomal proteases (Cathepsin S, L, K, B, D, E, H, and O) with protease inhibitors, compared to macrophages (Mφ), both in vivo (spleen and lymph node) and in vitro (BMDCs and BMMφ) [96]. Additionally, asparagine endopeptidase was also lower in BMDCs, as compared to BMMφ [96].

DCs also delay the maturation of endocytic compartments. In fact, the maturation kinetics of the phagosome occur later in BMDCs than in BMMφ [97], and the phagosomes and endosomes/lysosomes of BMDCs are kept under higher pH (7.5-8) conditions [98,99], in comparison to those of Mφ and neutrophils at pH 4.5-7 [99,100]. The high pH of the endocytic compartments is dependent on both the reduced activity of lysosomal V-ATPase [101] and the enhanced activity of nicotinamide adenine dinucleotide phosphate (NADPH) oxidase (NOX2) (Figure 1). 

The V-ATPase is a member of the ATP-dependent family of proton pumps and is ubiquitously localized on the membrane of intracellular compartments and the cell surface [102]. V-ATPase is composed of at least thirteen subunits, consisting of two domains: the V0 domain (a membrane-associated subunit) and the V1 domain (a periphery-associated subunit). The activity of V-ATPase is regulated by the assembly of the V0 and the V1 domains, which is under the control of innate immunity and mammalian targets of rapamycin (mTOR) in BMDCs [101,103]. The incomplete assembly of V-ATPase in DCs results in the protection of extracellular proteins from lysosomal degradation and an increase in CP efficiency [97]. It is well known that mTOR inhibitors, such as rapamycin, convert helper CD4^+^ T cells into regulatory CD4^+^ T cells by attenuating T-cell receptor (TCR) signals [104]. It may also be possible that mTOR inhibitors accelerate CP efficiency without an innate immunity signal, resulting in cross-tolerance by inducing CD8^+^ T cell anergy [29].

Nox2 catalyzes the production of reactive oxygen species (ROS) by transferring one electron to oxygen from NADPH. This occurs at very high rates (mM/s) and typically eliminates pathogens [105]. However, in DCs, ROS reacts with the protons in the luminal space, which causes an active alkalization [99,106,107]. NOX2 is made up of six subunits: Rac1 or Rac2, gp91phox (containing heme), p22phox, p40phox, p47phox, and p67phox [107]. Active alkalization by NOX2 was shown to be regulated by Rab27a [106], a plasma membrane SNARE protein called VAMP-8 (in both BMDCs and human moDCs) [108], phagosomal SNARE syntaxin-4, and SNAP-23 (in BMDCs) [109]. Rac2 regulated the recruitment and assembly of NOX2 in cDC1s, but not in cDC2s [99]. Deletion of the Wiskott-Aldrich syndrome protein (WASp) increased Rac2 activity, which resulted in enhanced CP efficiency, both in cDC1s and cDC2s [110]. In contrast, the reduced activity of either gp91phox or p47phox impairs the CP ability of BMDCs [99]. In cDC1s, sialic acid-binding immunoglobulin-type lectin-G (Siglec-G), a member of the lectin family, recruits Src homology region 2 domain-containing phosphatase-1 (SHP-1) to dephosphorylate p47phox, which inhibits NOX2 activation in phagosomes [111]. NOX2-defective DCs show impaired CP efficiency [99,111], which strongly suggests the important role of ROS production in CP. 

These low acidification states of endocytic compartments are inexpedient for DC homeostasis as they are essential to deactivate and degrade endocytosed pathogens, including many kinds of viruses [112,113,114]. In a normal state, acidification of the endocytic compartments is essential for self-protection of DCs from pathogens. Unaffected endocytic compartments are fused with the lysosome and show acidic conditions in fully mature DCs [101,111], in which the CP efficiency is reduced [115]. Since pathogen infections are avoidable with DC vaccination, pharmacological inhibitors of endocytic acidification (i.e., chloroquine and ammonium chloride) and lysosomal protease inhibitors (i.e., leupeptin) accelerated CP in human moDCs and cDC1s [116,117,118] and murine BMDCs [119], indicating that these pharmacological inhibitors may prove beneficial.

In BMDCs, extracellular proteins are internalized by fluid-phase pinocytosis or scavenger receptor-mediated endocytosis and are rapidly transported into a proteolytic endosome, where they are efficiently degraded by lysosomal proteases, resulting in low CP efficiency [120]. In contrast, the same extracellular proteins, simultaneously internalized by the mannose receptor (MR), are transported into a less-proteolytic endosome, where they are protected from lysosomal proteases, showing higher CP efficiency [120]. Similarly, in human moDCs, a less-proteolytic endosome for higher CP efficiency and a proteolytic endosome for lower CP efficiency operate side-by-side [118,121,122] (Figure 1). These two kinds of endosomes are also observed in human LCs; the langerin-bound extracellular proteins, which show effective CP ability, are transported into less-proteolytic endosome. In contrast, the dectin-1-bound extracellular proteins, which show nominal CP ability, are transported into proteolytic endosomes [69]. In addition, the inhibition of lysosomal protease in human moDCs results in effective CP of extracellular proteins once they are transported into the proteolytic endosome [118], indicating that maturation rates differ between these two endosomes. The less-proteolytic endosome may be responsible for preparing the surroundings for extracellular proteins to undergo ERAD-dependent processing (Figure 1). In DC vaccination, the accumulation of immunized proteins into this less-proteolytic endosome might accelerate CP efficiency. Since the molecular mechanisms of these distinctions are not clarified yet, further investigations are required to put these compartments to practical use in DC vaccinations. 

#### 3.2.2. The Non-Classical Endosome

The cytoplasmic delivery of exogenous proteins largely enhances CP efficiency [89], indicating that the retro-transport of internalized protein from endocytic compartments to the cytosol is one of the rate-limiting processes for an efficient CP. The intracellular transport pathways of extracellular proteins are different among DC subsets, such as cDC1s, moDCs, or BMDCs. The sources of exogenous proteins (e.g., soluble proteins, bead-bound proteins, or proteins expressed by heat-killed microbes) and their uptake routes (e.g., receptor-mediated endocytosis, pinocytosis, or phagocytosis) also vary between these subsets. Additionally, the particle size of the antigen-loaded beads influences the intracellular transport pathways [79]. Regardless of the multiple transport pathways, it is generally assumed that the ERAD machinery contributes to the translocation of extracellular proteins through the cellular membrane to the cytosol, and the ability of this retro-transportation is proportional to the varying CP efficiencies among different DC subsets [123]. While the exact molecular mechanisms of recognition and retro-transport have not been elucidated, extracellular proteins are transported into the cytosol and processed by the UPS [93,94,95] (Figure 1). ER-resident molecules, including the ERAD machinery, were also found in the phagosome of both Mφ [124,125] and cDCs [126]. The expression level of ERAD-related molecules (i.e., calreticulin, calnexin, SEC61α, SEC61β, SEC61γ, and PDIs) was higher in cDC1s than in cDC2s [44]. While ERAD is carried out in the ER, recent investigations have demonstrated that the ERAD-dependent processing in CP was brought about in the non-classical endocytic compartments, which contained both the ER-resident molecules and the endosome-specific molecules [93,94,95] (Figure 1). A purified subcellular compartment from moDCs, in which exogenous proteins undergo ERAD-dependent processing in vitro, contained both ER-resident proteins and endosome-specific proteins, as well as the precursor and mature forms of LAMP1 (before and after modification in the Golgi apparatus) [95]. Although the exact mechanisms of transport of ER-resident molecules are not completely understood, recent studies have shown that endosomes directly interact with the ER through the ER-endosome membrane contact site [127] or the ER-Golgi intermediate compartment (ERGIC) [128]. The two organelles exchange a wide variety of molecules during their maturation step. While the precise molecular mechanism of these transport pathways is not clarified yet, it is known that the SNARE proteins Sec22b (in the ERGIC) and syntaxin 4 (in the phagosome) regulate membrane fusion between the ERGIC and the phagosome [125,129]. However, later investigations into the role of Sec22b in CP showed contradictory results [130,131,132]; hence, the exact role of Sec22b in CP requires further examination.

It is not determined yet whether these non-classical endocytic compartments correspond to the less-proteolytic endosome. It might be possible that incorporated proteins are kept in the less-proteolytic endosome, and transport of ERAD-related molecules into these compartments enables ERAD-dependent processing of extracellular proteins before degradation in the lysosome. This non-classical endocytic compartment would play a critical role in DC vaccination. Further investigation is required to clarify the molecular mechanism that shapes this membranous compartment.

#### 3.2.3. Recognition of Extracellular Proteins as ERAD Substrates

As some extracellular proteins in ERAD-dependent processing are derived from infectious pathogens, infected cells, apoptotic cells, or cancers, their activities must be suspended after incorporation into DCs. Furthermore, extracellular proteins are clearly distinguished from endogenous proteins, because the processing of endogenous proteins not only consumes cellular resources, but also results in direct presentation (DP), which competes with CP. However, it is reported that extracellular proteins, such as gelonin (a membrane-impermeable ribosomal inhibitor) or cytochrome C, exhibit their function after retro-transport into the cytosol and inhibit the cellular functions in DCs or Mφ [69]. In general, since retro-transport of cytochrome C from incorporated cells does not induce the apoptosis of DCs, we can infer that retro-transport and degradation of extracellular proteins are tightly linked to each other.

**Figure 2:**
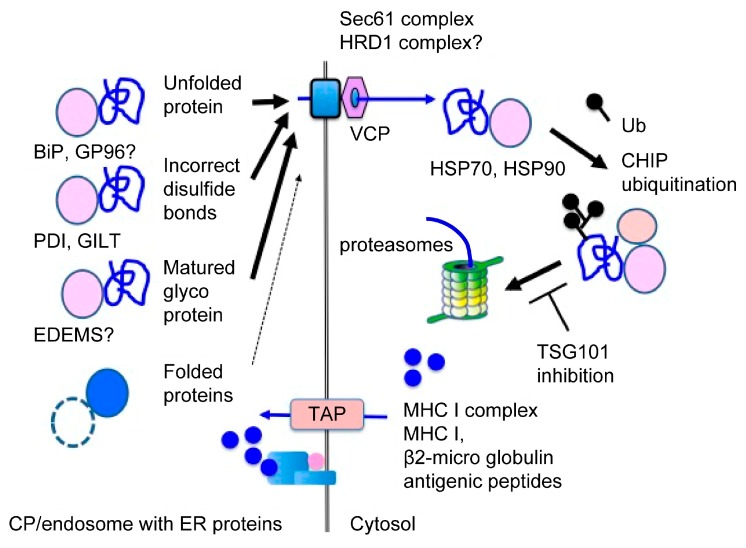
Recognition of extracellular proteins in CP. In ERAD-dependent processing, extracellular proteins are unfolded by ROS, resulting in nonspecific disulfide bonds in endocytic compartments. These unfolded extracellular proteins lose their activity and are preferentially recognized as ERAD substrates by ER-resident molecular chaperones, such as BiP, PDI, or ER-degradation enhancing α-mannosidase-like proteins (EDEMs). After recognition, these proteins are retro-transported into the cytosol. In contrast, self-proteins, which are properly folded in these cellular compartments, are not recognized as ERAD substrates and remain in endocytic compartments. After retro-transportation into the cytosol, extracellular proteins are recognized by cytosolic molecular chaperones, such as Hsp70 or Hsp90. After recognition, extracellular proteins are processed by the ubiquitin-proteasome system (UPS) into antigenic peptides and transported into membranous compartments in a transporter associated with antigen processing (TAP)-dependent manner. These two recognition steps indicate that unfolding and recognition of extracellular proteins are critical for CP.

Interestingly, extracellular proteins unfold [133] and specifically associate with ER-resident molecular chaperones in moDCs [134]. While the precise transport pathways of exogenous proteins in CP are unknown, the abovementioned results support the idea that extracellular proteins are unfolded on the transport pathway to the non-classical endosome [134]. This unfolding, which is also observed in the substrates of ERAD, distinguishes extracellular proteins from intracellular proteins (Figure 2) [59]. Additionally, unfolded proteins lose their activity. Since DCs maintain endocytic compartments under an alkaline pH (7.5–8) [99], exogenous proteins would not unfold under these conditions. As a means of inducing unfolding, DCs produce ROS to reduce the protons in endocytic compartments. ROS are oxidizing reagents that promote the formation of nonspecific disulfide bonds, resulting in the unfolding of extracellular proteins. In this context, extracellular proteins are specifically associated with PDI in mouse moDCs [134] and gamma-interferon-inducible lysosomal thiol reductase (GILT)—the only known thiol reductase localized in the lysosomes and phagosomes—which is essential for CP in mouse BMDCs (Figure 2) [135]. This suggests a critical role of disulfide bond formation in the unfolding, inactivation, and identification of extracellular proteins in CP. It is also possible that artificial disulfide bond formation in extracellular proteins increases CP efficiency. Recent research has shown that PDI inhibitors induce cancer-specific apoptosis in addition to the activation of cancer-specific T cells and the expansion of memory T cells in a mouse model [136]. In this experiment, cancer-specific T cells were activated by the addition of cancer-specific peptides; therefore, this is not the result of the progression of CP efficiency. Since PDI inhibition increases the recognition of extracellular proteins as substrates of ERAD-like processing, the additive effects seem encouraging.

In addition to PDI, extracellular proteins are also associated with ER-resident molecular chaperones in mouse moDCs, such as BiP, calreticulin [134], GP96, and ER-degradation enhancing α-mannosidase-like proteins (EDEMs) (our unpublished data). BiP and GP96 recognize unfolded proteins in the ER and play an important role in the retro-transport of these proteins in ERAD (Figure 2) [137]. The enhanced CP efficiency of BiP-bound antigens suggests that BiP plays an important role in CP [17]. Moreover, Hsp-complex proteins show high CP efficiency, supporting the hypothesis regarding the critical role of unfolding in CP [18,19]. The results from these mouse models suggest that unfolded extracellular proteins, associated with Hsp, are beneficial to the effects of DC vaccination. Additionally, Hsps are recognized as danger signals, which accelerate the maturation of DCs [18,19].

Exogenous proteins, which bind to the mannose receptor (MR, CD206, or MRC1) [120,138], langerin (CD207) [69], CD205 [5], or CLEC9A [139,140], showed high CP efficiency. Since all these receptors recognize sugar chain structures [65,141], it is possible that these lectin-bound proteins are also recognized as glycosylated ERAD substrates by ER-resident lectins, increasing CP efficiency. Conversely, MR-deficient BMDCs from mice showed poor CP ability for glycosylated proteins, which also supports this hypothesis [138]. In CP, large amounts of exogenous proteins are mature glycoproteins with high numbers of mannose-type oligosaccharide chains, which are preferentially recognized by ER-resident lectins as substrates for ERAD (Figure 2) [142]. Thus, it is possible that mannose-type oligosaccharide chains function as signals for exogenous proteins. While sugar chains play an important role in ERAD, the role of sugar chains in CP is not understood well. A recent investigation suggested that optimal glycosylation of antigenic proteins in vitro might serve as a useful adjuvant for DC vaccination [143].

#### 3.2.4. The Translocon in CP

Following recognition in the non-classical endocytic compartment, extracellular proteins are retro-transported through the cellular membrane into the cytosol. Together with retro-translocation, the extracellular proteins are ubiquitinated and processed by the UPS. Two retro-translocons are described in the ERAD: the HRD1 complex [144] and the Sec61 complex [145,146]. During retro-transport of CP, accumulating evidence suggests that the Sec61 complex plays a significant role. SEC61 α and β are associated with exogenous proteins in moDCs and BMDCs [134]. Inhibition of SEC61 by siRNA impairs CP ability in BMDCs [125], moDCs [126,134], cDC1s, and cDC2s [94]. Additionally, transport of the SEC61 complex into the endosome strongly inhibits CP in cDC1s, but not in cDC2s, indicating that extracellular proteins are retro-transported into the cytosol through the SEC61 complex and that the ERAD-dependent processing in CP was not carried out in the ER or the classical endosome, but in a non-classical endosome with ER-resident molecules [94]. Noticeably, exotoxin A, which binds the cytosolic N-terminal domain of Sec61 α and a nearby channel pore [147,148,149], inhibits the retro-transport of extracellular proteins to the cytosol both in vivo and in vitro [94,150], strongly indicating that the Sec61 complex plays a major role in the retro-transport of extracellular proteins in CP. It is possible that the substrates of the ERAD-dependent processing in CP are preferentially exported through the SEC61 complex, since they are simple, unstructured proteins without a transmembrane domain [151]. Pharmacological inhibition of the valosin-containing protein (VCP) and SEC61 recruitment by PYR-41 or thalidomide-mediated NF-κB inactivation decreased CP efficiency in BMDCs [152]. In contrast, in both BMDCs and moDCs, the retro-transport of extracellular proteins is independent of Derlin-1 [94,95], which is a component of the HRD1 complex [144]. Additionally, siRNA-mediated depletion of HRD1 in BMDCs impaired CP efficiency slightly, together with a robust impairment of MHC II presentation, suggesting that these results are nonspecific effects related to the downregulation of Hrd1 [94]. Nevertheless, the role of the HRD1 complex in CP has not been characterized as precisely as that of the SEC61 complex. It is reasonable to speculate, however, that the HRD1 complex may exert an effect on CP. In fact, our group also detected components of the HRD1 complex in a purified microsome for CP (personal unpublished data). This suggests that DCs may utilize different retro-translocon machinery for CP, such as the SEC61 complex and the HRD1 complex, depending on the condition of the exogenous proteins. However, further investigation is required to confirm this assumption. 

Only a small number of molecules have been shown to play a role in CP, compared to the retro-transport machinery. VCP was specifically associated with exogenous proteins in moDCs and BMDCs [134], and its inhibition abrogated CP ability in these DC subsets [95,134,138,150], indicating that p97 provides the energy to pull lumenal proteins into the cytosol. Similarly, carboxyl terminus Hsp70/90 interacting protein (CHIP), an E3 ubiquitin ligase, associated with CP substrates and played an essential role in CP in moDCs [134]. Tumor susceptibility gene 101 (TSG101), which is a dominant-negative regulator of polyubiquitination [153], colocalized with CP substrates and negatively regulated CP efficiency in BMDCs [138]. Cytosolic molecular chaperones, such as Hsp70 and Hsp90, were associated with CP substrates after retro-transport and were required for effective CP [133,154,155]. Altogether, these results indicate that ERAD-related molecules also play an important role in CP, suggesting that other ERAD-related molecules are also shared in CP. However, excessive ER stress results in inflammation or an immunosuppressive phenotype, which promotes cancer escape from the immune system. Further investigation is necessary to apply ERAD-dependent processing to antigen-loading methods. 

## 4. Peptide Loading onto MHC I

After processing, precursor peptides are trimmed to a suitable length of approximately 10 amino acids by cytosolic aminopeptidase (tripeptidyl peptidase 2; TPP2) [156] and are transported through the TAP complex into the ER or the non-classical endosome [80,81,125,127,154,157]. After transport into lumenal compartments, precursor peptides are further trimmed to a fitting length for MHC I presentation by ER-resident amino-peptidases (ERAP1 and ERAP2) [158,159] or insulin-responsive aminopeptidase (IRAP) [158,159,160]. This indicates that precursor peptides are transported into the ER and the endosomes and are then loaded onto MHC I [159]. Moreover, IRAP is localized in the Rab14-positive endocytic compartments, which further suggests that peptide loading may occur in the non-classical endocytic compartments [159,161]. 

The endocytic compartment is equipped with two kinds of MHC I molecules; one is a newly synthesized MHC I molecule without an antigenic peptide [80,81,108,111,125,135,149,162], and the other is recycled MHC I from the cell surface with antigenic peptides [130,163]. Newly synthesized MHC I are loaded with an antigenic peptide with the aid of PLC as DP, which ensures the variation of antigenic peptides. Alternatively, recycled MHC I are loaded with antigenic peptide by the peptide exchange reaction, in which old antigenic peptides are released upon recycled MHC I in the acidic compartments of the endosome/lysosome and are substituted by antigenic peptides [83,84]. In the peptide exchange reaction, the antigenic peptides with higher affinities for MHC I are preferentially selected. As a result, the variety of antigenic peptides decreases. The non-classical endosome is not as acidic as the endo/lysosome, so this compartment is unable to carry out an efficient peptide exchange reaction. Additionally, the non-classical endosome is equipped with MHC I and PLC [93]. Together, these results strongly suggest that peptide loading in CP is carried out by newly synthesized MHC I in the ER or in the non-classical endocytic compartments, which also supports the notion that the effective transport of extracellular proteins into the non-classical endocytic compartments is one of the most important requirements for DC vaccination. While the source of MHC I has not been precisely determined yet, the results of cytoplasmic delivery of exogenous proteins [89] indicate that the amounts of MHC I in DCs are sufficient, and that loading onto MHC I is not a rate-determining step in CP. 

## 5. Improvements in CP Efficiency by ERAD-Dependent Processing 

Since ERAD and the ERAD-dependent processing in CP share several cellular machineries, the molecular machinery of ERAD would be useful to improve CP efficiency. Notably, the recognition of extracellular proteins as the ERAD substrate would improve the retro-transport of extracellular proteins, a rate-limiting step of CP, which may contribute to the success of DC vaccinations. As previously mentioned, ex vivo methods, which enhance recognition of extracellular proteins by ER-resident molecular chaperones, would significantly improve the effects of DC vaccinations. It is also possible that the properties of extracellular proteins (unfolded, hyper-disulfated, or glycosylated) are one of the keys for effective DC vaccination (Figure 1). 

While activation of the non-canonical UPR is essential for CP in cDC1s [164], overactivation of the UPR in DCs sometimes has the opposite effect [164,165]. Although ROS play an essential role in antimicrobial defense [105] and take part in CP by actively alkalizing the luminal space [99,106,107] or by the membrane rupture pathway [92], ROS oxidize the cellular membrane and produce peroxidized lipids. In cDCs, dysregulated activation of UPR is induced by peroxidized lipids, leading to aberrant triglyceride synthesis and blunt anti-cancer immunity [165]. The oxidized lipids also ligate TLR4 and cause inflammation [166,167]. Additionally, in cDC1s, overactivation of the UPR resulted in decreased expression of tapasin and hampered CP [164]. In murine herpes simplex virus (HSV) type 2 infection experiments, viral glycoprotein D increased ER stress and inhibited functions of BMDCs, such as migration and initiation of naïve T cells [168]. This also supports the notion that the overactivation of UPR impairs both the antigen presentation and the immunoregulatory activity of DCs. Cancer UPR can induce UPR in cancer-infiltrating immune cells extrinsically, an action called transmission of ER stress (TES) [62,169]. TES imparts BMDCs with a mixed pro-inflammatory/immunosuppressive phenotype to promote cancer survival and outgrowth, although the precise molecular mechanisms are not clarified yet [62]. Moreover, during TES, monocytes differentiate into MDSCs, which accumulate in cancers and block protective anti-cancer T cell responses in both humans and mice [63]. All of these results indicate that the molecular machinery of ERAD-dependent processing has great potential to increase CP efficiency, and further investigation is essential to make good use of this for DC vaccines.

## 6. DC Maturation

CP efficiency is one of the most important factors in DC vaccination, as CTLs are not only activated by peptide-loaded MHC I. To induce CTLs fully, costimulatory molecules (CD40, CD80, and CD86) on the DC surface are essential [4,5,6,7]. Additionally, to preserve cancer-specific CD8^+^ T cells, sufficient cytokines from helper T cells are required to expand CTLs into effector-memory CD8^+^ T cells [41]. Effector-memory CD8^+^ T cells play a crucial role in long-lasting cancer immunity and immunosurveillance for cancer redevelopment [170,171]. While cDC1s and moDCs are capable of effective CP in steady state, CP without costimulatory molecules and appropriate cytokines induces T cell cross-tolerance, which plays a significant role in suppression immunotherapy [7]. DCs express these costimulatory molecules and key cytokines only after activation by ligation of PRRs [172,173] or receiving several kinds of cytokines [174,175]. After detecting stimulation, DCs undergo a developmental program called maturation, transitioning from immature antigen-capturing DCs to mature antigen-presenting DCs, accompanied by morphological changes [176]. DCs migrate into draining lymph nodes to shape the fittest adoptive immune response according to stimulation by PRRs or cytokines [176]. Some immune response modifiers, such as imiquimod [177], BCG, or picibanil [178], which are approved as anti-cancer drugs, activate innate immunity and facilitate DC activation and maturation. These results indicate that the activation of innate immunity plays an essential role in activation immunotherapy, including DC vaccination.

Maturation transiently enhances the incorporation of extracellular proteins [179], thereby increasing CP efficiency [176]. The ligation of TLR4 in BMDCs promotes CP by several molecular mechanisms. First, internalization of extracellular proteins is increased [115]. Second, phagosome/endosome maturation is delayed, which protects extracellular proteins from degradation by the lysosome [180]. Additionally, the activation of TLR4 downregulates vacuolar proton ATPase, cathepsin B, D, S, and Rab7 [97,180,181] and upregulates MHC I, PLC, and UPS molecules [181]. The activation of either TLR2 or TLR4 accelerates the fusion between phagosomes and MHC I-containing recycling endosomes [130,182]. In cDC1s, TLR3 stimulation enhances the uptake of apoptotic cells [183], and stimulation of NOD1 and NOD2 accelerates CP by upregulating PLC and ERAD-related molecules [184]. In human moDCs, NODs and TLR2 stimulation enhanced CP by positively regulating MHC I peptide loading and immunoproteasome stability [185]. To the contrary, neither TLR4, Myd88, nor TRIF-deficient BMDCs showed decreased CP efficiencies in mice [159]. Extracellular proteins in endocytic compartments without danger signals are self-proteins; therefore, they are promptly degraded in the lysosome, resulting in lower CP efficiency (Figure 1). In contrast, extracellular proteins with danger signals would be non-self-proteins; hence, they undergo ERAD-dependent processing and show higher CP efficiency (Figure 1). These results suggest that the activation of innate immunity may cause delayed maturation of endocytic compartments with non-self-proteins [118,119,120,121,122], resulting in improvements to the CP efficiencies of non-self-proteins in addition to the induction of DC maturation.

After the transient augmentation of extracellular protein incorporation, DCs shut the incorporations down, and CP efficiencies are finally depressed [176]. In BMDCs and cDC1s, expression of TFEB, a lysosomal master regulator activated by the innate immunity signal [186], negatively regulates CP ability by upregulating lysosomal proteases and promoting the maturation of lysosomes [111]. TFEB is reportedly activated by NOX2 lysosomal alkalization and, when activated, directly initiates transcription of costimulatory molecules [187]. Additionally, in mice, TFEB controls transcription of several cytokines and chemokines, including IL- 1β, IL-6, TNF-α, and CCL5 [186], indicating that activation of TFEB controls the maturation steps of DCs. After receiving an innate immunity signal, DCs inhibit the maturation of endocytic compartments by several molecular mechanisms including ROS production and retro-transport of extracellular proteins into the cytosol for CP. Then, ROS activate TFEB, and TFEB promotes the maturation of lysosomes that exterminate pathogens [111] and starts the transcription of costimulatory molecules and cytokines for cross-priming [186,187]. The accelerated activation of V-ATPase after the ligation of TLR4 in BMDCs [101] is inconsistent with the results from the previously mentioned effects of innate immunity, and is the result of the time difference in the maturation of the lysosome (Figure 1). The role of TFEB in CP indicates that the maturation step of DCs is another rate-limiting step for CP, which strongly suggests the importance of the time sequences of DC maturation for DC vaccination, particularly the time interval between administration of extracellular proteins and maturation signals. 

In addition to CP efficiency, the maturation status of DCs influences the selection of the administration route. Under non-artificial conditions, DCs incorporate antigenic proteins in the tumor site, start maturation, and migrate into the T cell-rich zone of draining lymph nodes to induce an immune response by activating tumor specific CTLs [188,189]. The tumor site resident cDC1s secrete CXCL9/10 chemokine to induce these tumor-specific CTLs [12]. These specializations (antigen incorporation, cross-priming, and chemokine secretion) are determined by the localizations and the maturation status of DCs [190]. However, both lymph nodes migrated cDC1s and the tumor site resident cDC1s play significant roles in anti-tumor immune response [11], the maturation status of DCs and administration route (intranodal, intradermal, intravenous, intralymphatic, etc.) were uniformly regulated in DC vaccination. It was recently reported that migration of DCs into tumor nests was under development as a human cancer therapy, but without significant success [191]. These results strongly suggest that selection of the administration route, in consideration of the maturation status of DCs, is one strategy to overcome the obstacles associated with DC vaccination. 

All these results indicate that DC maturation is critical to the effect of DC vaccination, since the maturation step has determinative effects on cross-priming efficiency, the expansion of effector-memory CD8^+^ T cells, and the infiltration of activated CTLs, all of which play important roles in tumor immunity.

## 7. Conclusions

Antigen presentation, especially by CP, is one of the most important rate-determining steps for DC vaccination and suppression immunotherapies. Despite the essential role of CP in an adaptive immunity, its precise molecular mechanism is not yet characterized. The intracellular transport pathways of extracellular proteins differ among DC subsets, depending on the properties of exogenous proteins and their route of uptake. Despite these differences, recent research has revealed that limited lysosomal degradation and ERAD-dependent processing play pivotal roles in the production of the antigenic peptide from extracellular proteins in CP. The role of ERAD-dependent processing in CP strongly suggests that ex vivo methods, which enhance recognition of extracellular proteins as the ERAD substrate, would significantly improve the effect of DC vaccination. On the contrary, the overactivation of UPR impairs both antigen presentation and the immunoregulatory activity of DCs, and even differentiates monocytes into MDSCs or MDSC-like cells [62,63]. This indicates that the application of ERAD-dependent molecular machinery may have extensive potential to improve CP efficiency. To this end, our laboratory is purifying subcellular compartments in which exogenous antigens undergo ERAD-dependent degradation by DCs in different cell subsets, uptake routes, or maturation statuses, according to our previous methods [93]. These results will clarify the detailed molecular mechanisms of CP and may contribute to the improved design of DC vaccines. Further studies are needed regarding the molecular machinery of non-canonical UPR to optimize it for DC vaccines. Additionally, the time sequences between the administration of extracellular proteins and the maturation signals should be appropriately arranged considering the maturation step of DCs, which regulates ERAD-dependent processing. In conclusion, accumulating evidence suggests that the molecular mechanisms of ERAD-dependent processing can be applied to improve the efficacy of DC vaccination, but not before the precise role of ERAD-dependent processing in CP is determined.

## Figures and Tables

**Table 1:** Major dendritic cell (DC) subsets and surface markers [70,71].

DC subsets	Mouse		Human	
	Surface Marker	Cytokine Profile	Surface Marker	Cytokine Profile
cDC1	CD11c, MHC II, CD8α, CD103, CD24, XCR1, CLEC9A, CD205	IL-12 (high), IFN-III, IFN- λ	CD11c (low), HLA-DR, CD141, CD205, CLEC9A, XCR1, Nec12	IL-12 (low), IFN-III, IFN-λ
cDC2	CD11c, MHC II, CD11b, CD172a (Sirpα), ESAM	IL-6, TNF	CD11c, HLA-DR, CD1c, CD11b, CD172a (Sirpα), CD1a, CD14, CD5	IL-12, IL-1β, TNF, IL-6, IL-10, IL-23, IFN-γ
pDC	CD11c, MHC II, B220, CD317, SIGLEC-H, CD172a, CD209, CCR2, CCR9, CXCR3	Type I and III IFN	CD11c (low), HLA-DR (low), CD123, CD303, CD304, CCR2, CXCR3	Type I and III IFN
moDC	CD11c, MHC II, CD11b, CD172, F4/80, Ly6C, CD64 (FcεRI)	IL-12, IL-23, IL-6, IL-10	CD11c, HLA-DR, CD1c, CD1a, CD11b, CD172a, CD64 (FcεRI), CD14, CD5, CD206	IL-12, IL-23, IL-6, IL-10
LC	CD11c, CD1d, CD207 (langerin), E-cadherin, MHC II, CD205		CD11c, CD1a, CD1b, CD1c, CD207 (langerin), E-cadherin, HLA-DR, CD205	
